# Anatomical lymphatic drainage basin and sentinel lymph node positivity in Merkel cell carcinoma: a 37-year single-center cohort study of 400 patients

**DOI:** 10.3389/fonc.2026.1900347

**Published:** 2026-08-12

**Authors:** Maximilian Kueckelhaus, Entela Kupina, Laetitia Chiarella, Joel Mittelbach, Filipa Almeida Oliveira, Tobias Hirsch, Hans-Joachim Schulze, Jan Dirk Raguse, Grit Sophie Herter-Sprie, Sascha Wellenbrock

**Affiliations:** 1 Department of Plastic, Reconstructive and Aesthetic Surgery, Hand Surgery, Fachklinik Hornheide, Münster, Germany; 2 Department of Plastic Surgery, University Hospital Münster, Münster, Germany; 3 Department of Plastic and Reconstructive Surgery, Institute for Musculoskeletal Medicine, University Hospital Münster, Münster, Germany; 4 Department of Dermatology, Dermatological Radiotherapy and Dermatohistopathology, Fachklinik Hornheide, Münster, Germany; 5 Department of Oral and Maxillofacial Surgery, Fachklinik Hornheide, Münster, Germany; 6 Department of Medical Oncology, Fachklinik Hornheide, Münster, Germany; 7 Faculty of Medicine, University of Cologne, Cologne, Germany

**Keywords:** anatomical drainage basin, completion lymph node dissection, Merkel cell carcinoma, nodal staging, occult nodal metastasis, sentinel lymph node biopsy

## Abstract

**Background:**

Merkel cell carcinoma (MCC) is a rare, aggressive cutaneous neuroendocrine malignancy with increasing incidence due to UV exposure and improved diagnostics. It spreads rapidly via lymphatics and has high mortality, surpassing melanoma in lethality. Management includes surgical excision, sentinel lymph node biopsy (SLNB), radiotherapy, and immunotherapy. SLNB is an established staging tool for detecting occult nodal metastases; however, the influence of anatomical drainage basin location on SLNB positivity and the role of completion lymph node dissection (CLND) remain incompletely understood. This study evaluated patterns of nodal involvement according to drainage basin location and assessed the diagnostic yield of CLND in a large single-center MCC cohort.

**Methods:**

A retrospective analysis was performed of 400 patients with MCC treated at a tertiary referral center between 1986 and 2023. SLNB positivity rates were compared across cervical, axillary, and inguinal drainage basins. Associations between anatomical location and nodal involvement were assessed using univariable and multivariable logistic regression models. Adjusted analyses included year of diagnosis, lymphovascular invasion and immunosuppression status, with Firth’s penalized regression applied where appropriate.

**Results:**

Among 400 patients, 244 underwent SLNB, of whom 71 (29.1%) had at least one positive SLN. In univariable analysis, inguinal SLNB was associated with higher odds of positivity compared with axillary SLNB (OR 3.31, 95% CI 1.49–7.35; p = 0.003) and cervical SLNB (OR 2.10, 95% CI 1.04–4.24; p = 0.040). After adjustment for lymphovascular invasion, immunosuppression, and year of diagnosis, inguinal drainage remained associated with SLNB positivity compared with axillary drainage (OR 3.08; 95% CI 1.36–6.98; p = 0.007). Lymphovascular invasion (OR 3.29; 95% CI 1.66–6.54; p = 0.001) and immunosuppression (OR 2.38; 95% CI 1.12–5.06; p = 0.025) were also associated with SLNB positivity in the adjusted model.

**Conclusions:**

SLNB remains a valuable staging procedure in MCC, identifying occult nodal disease in nearly one-third of patients. Inguinal drainage basin remained associated with SLNB positivity after adjustment for clinicopathological factors. These findings may help inform clinical decision-making regarding nodal staging in patients with MCC. The substantial rate of additional nodal involvement detected by CLND supports its continued role as a staging procedure in selected patients with MCC. As this study focuses on lymphatic dissemination assessed by SLNB and lymph node dissection (LND), no conclusions can be drawn regarding hematogenous metastatic spread.

## Introduction

**Figure 1:**
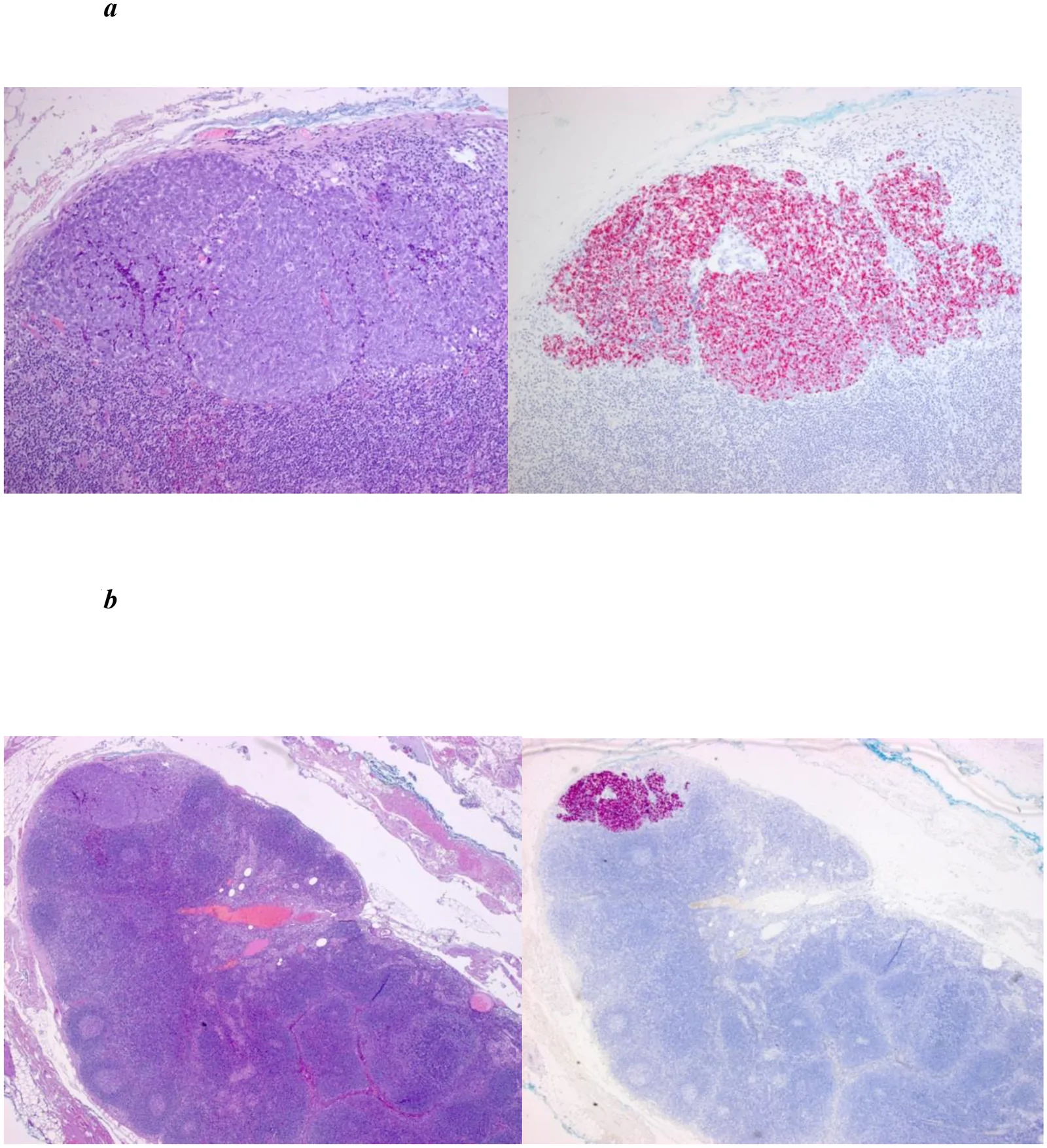
**(a)** Histopathological images of a primary Merkel cell carcinoma of the skin with and without H&E staining. **(b)** Histopathological appearance of a positive lymph node with Merkel cell tumor involvement, with and without H&E staining.

Merkel cell carcinoma (MCC) is a rare cutaneous neuroendocrine carcinoma that typically presents as a dermal or dermosubcutaneous neoplasm (see **Figure 1a**). Although incidence varies geographically, ranging from approximately 0.7 per 100,000 population in the United States to 2.5 per 100,000 in Australia, MCC remains an uncommon malignancy with distinctive clinical features (1–3).

MCC is characterized by rapid growth, early lymphatic spread, and a notable risk of recurrence (4–6). Factors such as increased life expectancy, cumulative UV exposure, improved diagnostic methods, and greater clinical awareness have contributed to a rise in reported incidence (2, 7). Although more recent epidemiological analyses suggest that incidence rates may have stabilized in some regions, the absolute number of MCC cases is expected to continue increasing due to population aging and demographic changes (8). The head and neck region is the most common site of MCC, followed by the upper and lower extremities and the trunk, reflecting its predilection for chronically sun-exposed skin (9).

The management of MCC has evolved considerably in recent years, favoring a multimodal approach. Core components include surgical excision with histologically clear margins, sentinel lymph node biopsy (SLNB) for accurate staging, adjuvant radiotherapy to reduce recurrence risk, and anti-programmed death (ligand)-1 immunotherapy for advanced disease (10, 11).

Among these treatment modalities, SLNB has become an integral component of nodal staging in clinically node-negative MCC (12–15). It is the standard procedure for detecting occult regional nodal metastases, providing essential staging information that guides subsequent treatment decisions (13, 15). However, little is known about whether SLNB positivity varies according to the anatomical lymphatic drainage basin and whether such differences persist after adjustment for established clinicopathological risk factors. Furthermore, the diagnostic yield of completion lymph node dissection (CLND) following positive SLNB remains incompletely characterized in large contemporary cohorts. Although MCC may also metastasize via the hematogenous route, the present study specifically focuses on lymphatic dissemination as assessed by SLNB and lymph node dissection (LND) (16).

Therefore, the present study aimed to evaluate the association between anatomical lymphatic drainage basin and SLNB positivity and to determine the prevalence of additional nodal metastases identified by CLND following a positive SLNB.

## Methods

**Figure 2:**
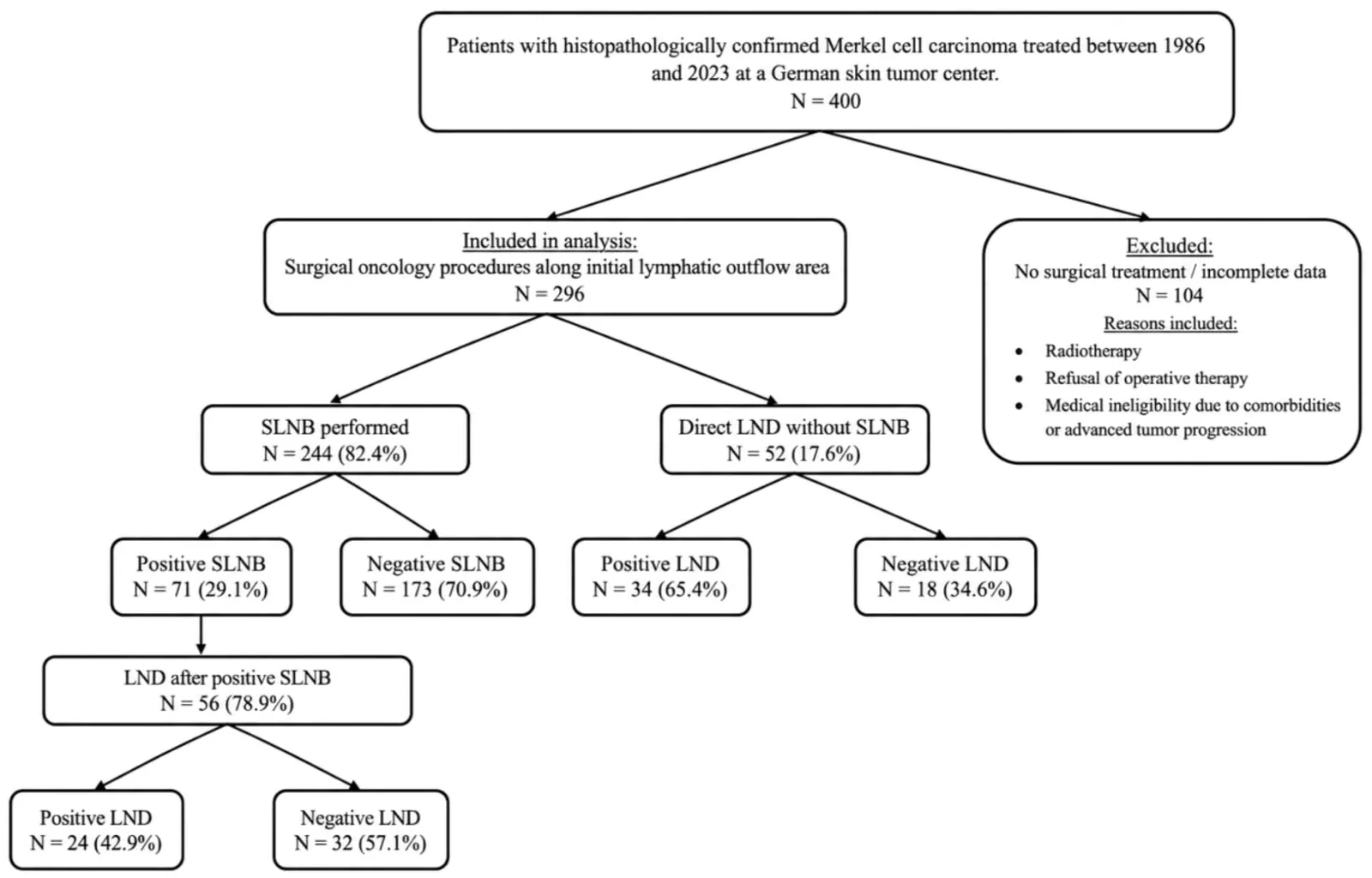
Patients selection flow chart.

This retrospective study, approved by the institutional review board (Medical Council Westfalen-Lippe, Number 2022-611-f-S), included 400 adult patients diagnosed with MCC at the Fachklinik Hornheide between 1986 and 2023. Patient data were extracted from the clinic’s database, focusing on those who underwent SLNB and/or LND. Patients without documented SLNB or LND procedures were excluded from the primary analyses, as illustrated in the study flow chart (**Figure 2**). Key variables included patient demographics, tumor characteristics, and details of SLNB and LND procedures (location and number of affected lymph nodes).

### Primary analysis

The main analysis focused on SLNB outcomes. The Fisher’s exact test was used to evaluate SLNB results across axillary, inguinal, and cervical regions. Univariable logistic regression determined the odds of a positive SLNB result by dissection location, with axillary tumor involvement as the reference group. SLNB positivity was defined as histopathological evidence of metastatic Merkel cell carcinoma in at least one SLN.

### Secondary analysis

The distribution of positive lymph node results across dissection locations (axillary, inguinal, cervical) was assessed using Fisher’s exact test. Logistic regression models evaluated the association between dissection location and positive lymph node findings. Lymph node positivity after LND was defined as histopathological detection of metastatic tumor in one or more additional non-SLN.

### Advanced cases

According to the German S2k guideline, patients with histopathologically confirmed MCC typically undergo SLNB as an initial step to evaluate potential lymph node metastasis^13^. For patients with advanced MCC, SLNB may be bypassed in favor of immediate LND. A subgroup analysis examined lymph node outcomes in this group, using Fisher’s exact test and logistic regression.

### Subgroup analysis of positive SLNB and CLND outcomes

Patients with positive SLNB results who underwent CLND were analyzed to compare lymph node positivity by dissection location. Fisher’s exact test and logistic regression were used to assess differences.

### Statistical analysis

All analyses were performed at the patient level. Each patient contributed only one anatomical nodal basin, defined by the location of the SLNB or, where applicable, the primary lymph node dissection. Consequently, no patient contributed more than one nodal basin to the analyses. Missing data were handled by complete-case analysis without imputation. No missing values were present among the covariates included in the multivariable model, so all 244 patients contributed to the analysis and complete-case analysis was equivalent to the full cohort.

Non-parametric tests (Fisher’s exact test) were used to compare group distributions. The association between SLNB positivity and anatomical location was first assessed with univariable logistic regression, and results are presented as odds ratios (OR) with 95% confidence intervals (CI) and p-values. To account for potential confounding, multivariable logistic regression models were constructed adjusting for lymphovascular invasion and immunosuppression status, with year of diagnosis included to account for temporal changes over the study period. Lymphovascular invasion (LVI), defined as the histopathological presence of tumor cells within lymphatic or blood vessels, was recorded from the original pathology reports. No central pathological review was performed, and no retrospective reclassification using standardized criteria was undertaken. LVI classification therefore relied on the contemporaneous pathology reports available at the time of diagnosis.

AJCC stage at treatment initiation was not included in the primary model because its higher categories incorporate nodal status determined by the SLNB, which would introduce circularity. In cases of collinearity or small-sample bias, Firth’s penalized regression was applied. Exploratory analyses were performed in the subgroup of patients with positive SLNB to evaluate subsequent LND outcomes across anatomical regions. Model discrimination was quantified using the area under the receiver operating characteristic curve (AUC). To account for overfitting, an optimism-corrected AUC was estimated by bootstrap validation with 500 resamples (Harrell’s method). The model was refitted in each bootstrap sample, its performance was measured both on the bootstrap sample and on the original cohort, and the mean difference (optimism) was subtracted from the apparent AUC. Calibration was assessed using the Hosmer–Lemeshow goodness-of-fit test; because Firth’s penalized estimator does not support post-estimation goodness-of-fit testing, calibration was evaluated on the corresponding maximum-likelihood logistic model fitted to the same covariates. The adjusted model comprised 71 outcome events for 5 estimated parameters (two drainage-basin indicators, lymphovascular invasion, immunosuppression, and year of diagnosis), corresponding to approximately 14 events per variable.

Statistical significance was defined as a two-sided p-value <0.05. The univariable comparisons in **Table 1** are descriptive and were not adjusted for multiple testing; they are therefore interpreted as hypothesis-generating. All analyses were performed with Stata (Version 16, StataCorp LLC, College Station, TX, USA).

**Table 1:** Differences in Patient and Tumor Characteristics between SLNB Drainage basin (N = 244).

Characteristic	Axillary (N = 79)	Inguinal (N = 46)	Cervical (N = 119)	P-value
Age at presentation, years	72.6 (± 9.6)	71.5 (± 9.4)	76.3 (± 10.1)	0.004
Female sex	29 (36.7%)	22 (47.8%)	68 (57.1%)	0.02
BMI, kg/m^2^	29.4 (± 5.1)	28.7 (± 5.8)	26.9 (± 4.4)	0.002
Remission/disease course				0.24
No	2 (2.5%)	0 (0.0%)	2 (1.7%)	
Yes	38 (48.1%)	17 (37.0%)	56 (47.1%)	
Unknown	33 (41.8%)	21 (45.7%)	39 (32.8%)	
Progressive	6 (7.6%)	8 (17.4%)	22 (18.5%)	
SLNB positive	16 (20.3%)	21 (45.7%)	34 (28.6%)	0.01
Tumor thickness, mm	10.1 (± 6.3)	9.5 (± 5.9)	8.7 (± 5.6)	0.48
Hospital stay, days	11.3 (± 10.5)	18.5 (± 28.3)	15.9 (± 10.2)	0.02
Tumor location				<0.001
Arms	72 (91.1%)	1 (2.2%)	0 (0.0%)	
Legs	0 (0.0%)	35 (76.1%)	0 (0.0%)	
Trunk	6 (7.6%)	6 (13.0%)	1 (0.8%)	
Head/Neck	0 (0.0%)	0 (0.0%)	117 (98.3%)	
Other	1 (1.3%)	4 (8.7%)	1 (0.8%)	
Asymptomatic	73 (92.4%)	43 (93.5%)	117 (98.3%)	0.11
Expanding rapidly				0.93
No	1 (1.3%)	1 (2.2%)	1 (0.8%)	
Yes	52 (65.8%)	28 (60.9%)	79 (66.4%)	
Unknown	26 (32.9%)	17 (37.0%)	39 (32.8%)	
UV exposure history				0.001
Yes	33 (41.8%)	8 (17.4%)	58 (48.7%)	
Unknown	46 (58.2%)	38 (82.6%)	61 (51.3%)	
Fitzpatrick skin type				<0.001
Unknown	15 (19.0%)	10 (21.7%)	13 (10.9%)	
I	15 (19.0%)	11 (23.9%)	65 (54.6%)	
II	44 (55.7%)	25 (54.3%)	35 (29.4%)	
III	5 (6.3%)	0 (0.0%)	6 (5.0%)	
Other cutaneous malignancy	20 (25.3%)	7 (15.2%)	46 (38.7%)	0.007
Complete AEIOU criteria	1 (1.3%)	1 (2.2%)	8 (6.8%)	0.13
>3 AEIOU criteria	62 (78.5%)	31 (67.4%)	100 (84.0%)	0.06
Ulceration	9 (11.4%)	2 (4.3%)	27 (22.7%)	0.007
Metastases				0.06
None	57 (72.2%)	21 (45.7%)	66 (55.5%)	
Lymph node	12 (13.9%)	15 (32.6%)	18 (15.1%)	
Satellite metastases	3 (3.8%)	3 (6.5%)	10 (8.5%)	0.43
In-transit metastases	6 (7.6%)	6 (13.0%)	24 (20.2%)	0.047
Lymph node dissection performed	15 (19.0%)	16 (34.8%)	44 (37.0%)	0.022
LND positivity	6 (7.6%)	9 (19.6%)	17 (14.3%)	0.14
Lymphovascular invasion	13 (16.5%)	10 (21.7%)	21 (17.6%)	0.75
Radiotherapy to primary region	50 (63.3%)	26 (56.5%)	89 (74.8%)	0.09
Chemotherapy	4 (5.1%)	5 (10.9%)	5 (4.2%)	0.24
Immunotherapy	4 (5.1%)	5 (10.9%)	8 (6.7%)	0.46
Immunocompromised	8 (10.1%)	8 (17.4%)	19 (16.0%)	0.42
Rheumatologic history	3 (3.8%)	4 (8.7%)	8 (6.7%)	0.51
Hyperuricemia	12 (15.2%)	5 (10.9%)	13 (10.9%)	0.64
Hypertension	67 (84.8%)	35 (76.1%)	92 (77.3%)	0.36
Diabetes mellitus	20 (25.3%)	13 (28.3%)	31 (26.1%)	0.94

Continuous variables are mean (SD); categorical variables are n (%). p-values from Fisher’s exact test (categorical) or Kruskal–Wallis (continuous).

## Results

### Patient demographics

A total of 400 patients were included in the study. Of these, 296 underwent surgical oncology procedures along their initial lymphatic outflow area, including 244 (82.43%) who received SLNB either alone or followed by CLND, while 52 (17.57%) underwent lymph node dissection without prior SLNB.

The mean age was 74.20 ± 9.97 years, and 48.77% were female, with a mean BMI of 28.07 ± 5.02. When stratified by lymphatic basin, patients with cervical SLNB were significantly older (mean age 76.3 ± 10.1 years) than those with axillary (72.6 ± 9.6 years) or inguinal SLNB (71.5 ± 9.4 years) (p = 0.004). Sex distribution also differed, with higher female proportions in the cervical group (57.1%) compared to axillary (36.7%) and inguinal (47.8%) (p = 0.019).

BMI varied across regions, with cervical patients having the lowest mean (26.9 ± 4.4) compared to axillary (29.4 ± 5.1) and inguinal (28.7 ± 5.8) (p = 0.002). Length of hospital stay was longest in the inguinal group (18.5 ± 28.3 days) compared with cervical (15.9 ± 10.2) and axillary (11.3 ± 10.5) (p = 0.024). Full details are presented in **Table 2**.

**Table 2:** Demographic data of patients having undergone SLNB and/or lymph node dissection.

	SLNB &/lymph-node dissection N = 244
Demographics	
Age*, years	74.20 ± 9.97
Sex (female)	119 (48.77%)
BMI*, kg/m^2^	28.07 ± 5.02
Skin type	
I	91 (37.30%)
II	104 (42.62%)
III	11 (4.51%)
Tumor localization	
Arms	73 (29.92%)
Legs	35 (14.34%)
Head/Neck	117 (47.95%)
Torso	13 (5.33%)
Unknown Primary	6 (2.46%)
Comorbidities	
Rheumatoid Arthritis	15 (6.15%)
Hyperuricemia	30 (12.30%)
Thyroid Pathologies	
*Hypothyroidism*	47 (19.26%)
*Hyperthyroidism*	3 (1.23%)
*Struma*	8 (3.28%)
*St.p. thyroidectomy*	5 (2.05%)
Hypertension	194 (79.51%)
Hypercholesterinemia	107 (43.85%)
Diabetes	64 (26.23%)
Smoker	15 (6.15%)

Data is reported in N (%) and mean ± standard deviation (SD) if not otherwise specified.

^§^ Relative percentage to total performed procedures.

Cervical patients also had more frequent ulceration (22.7% vs. 11.4% axillary, 4.3% inguinal; p = 0.007) and more in-transit metastases (20.2% vs. 7.6% axillary, 13.0% inguinal; p = 0.047).

Skin type distributions differed substantially: cervical patients were predominantly Fitzpatrick type I (54.6%), while axillary and inguinal groups were mainly type II (55.7% and 54.3%, respectively) (p < 0.001). A history of other cutaneous malignancies was also more common in cervical cases (38.7%) compared with axillary (25.3%) and inguinal (15.2%) (p = 0.01). Full details are presented in **Table 3**.

**Table 3:** SLNB and LND findings, stratified by procedure cohort (N = 244 SLNB; N = 75 CLND; N = 52 primary LND).

	SLNB (n = 244)	CLND after SLNB (n = 75)	Primary LND, no prior SLNB (n = 52)
Nodal positivity			
Positive, n (%)	71 (29.10%)	32 (42.67%)	34 (65.38%)
Nodes examined/involved			
Nodes removed, median (IQR)	2 (1–3)	11 (7–21)	13 (6–22)
Lymph-node metastasis size			
Micro, n (%)	31 (12.70%)	7 (9.33%)	3 (5.77%)
Macro, n (%)	19 (7.79%)	9 (12.00%)	18 (34.62%)
Micro & Macro, n (%)	5 (2.05%)	4 (5.33%)	5 (9.62%)
Diameter, mm, median (IQR)	2.45 (0.65 - 7.45)	5.42 (3.80- 12.00)	11.4 (8 – 19)
Extracapsular extension			
Capsule breach, n (%)	20 (8.20%)	11 (14.67%)	17 (32.69%)
Anatomical localization, n (%)			
Axillary	79 (32.38%)	15 (20.00%)	13 (25.00%)
Inguinal	46 (18.85%)	16 (21.33%)	10 (19.23%)
Cervical	119 (48.77%)	44 (58.67%)	29 (55.77%)

Data is reported in N (%) and mean ± standard deviation (SD) if not otherwise specified.

*Reported as median and Inter-quartile range (IQR).

### Sentinel lymph-node biopsy

**Figure 3:**
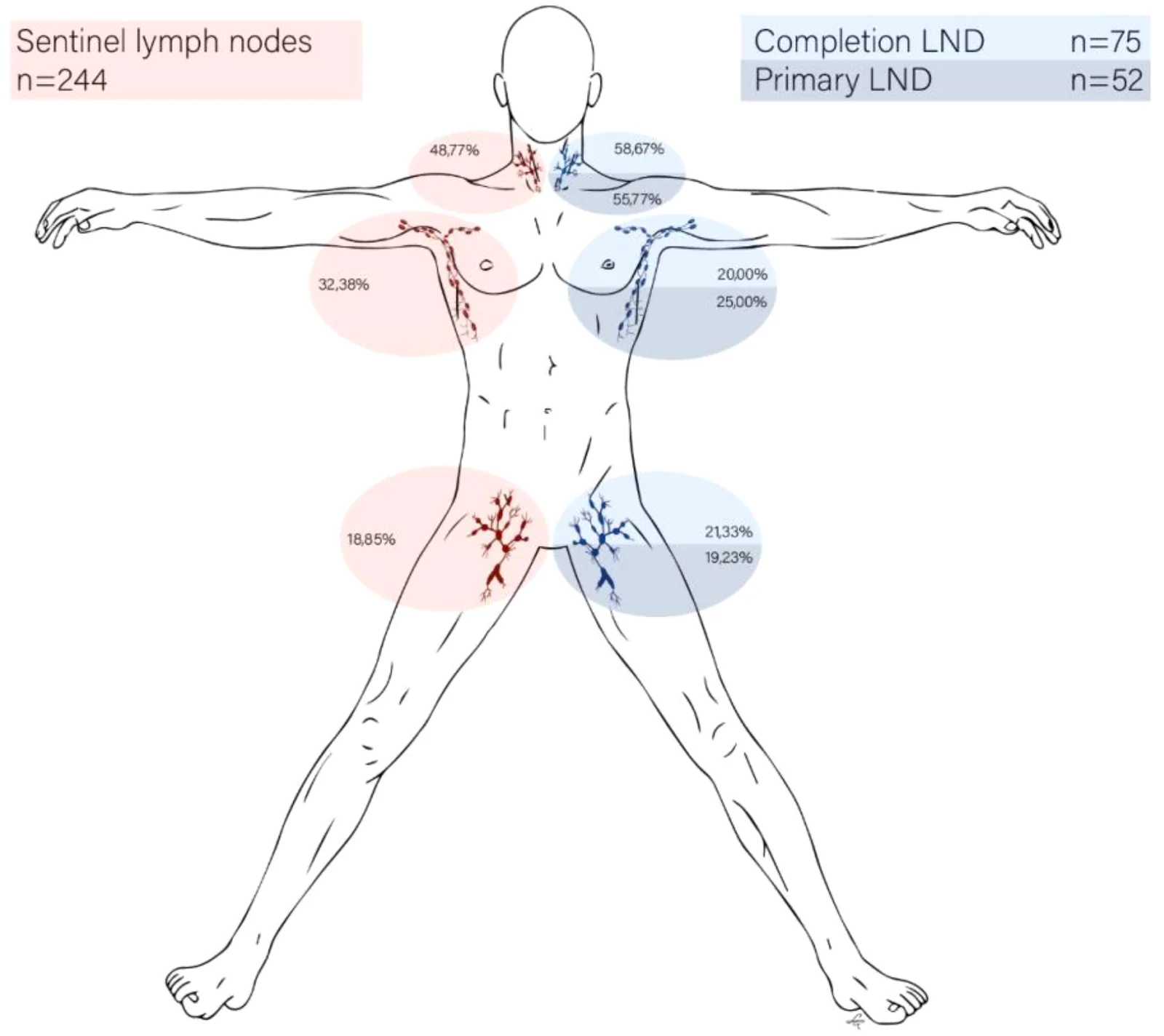
Schematic representation of lymph node involvement in SLNBs and LND across three anatomical regions: cervical, axilla and inguinal region. The left side of the figure indicates the distribution of SLNBs, with percentages representing the total biopsies taken. The right side illustrates the corresponding regions for LND reflecting the total dissections performed.

SLNB distribution and basin-specific SLNB positivity rate were first examined across anatomical regions. Of 244 SLNBs performed, 79 were axillary (32.38%), 46 inguinal (18.85%), and 119 cervical (48.77%) (**Table 3**; **Figure 3**). Overall, 71 patients (29.10%) had at least one metastatic SLN. The probability of identifying at least one metastatic SLN during SLNB differed significantly according to the anatomical drainage basin. Among patients undergoing SLNB, 45.7% (21/46) of those with an inguinal drainage basin had a positive SLNB, compared with 28.6% (34/119) in the cervical basin and 20.3% (16/79) in the axillary basin (Fisher’s exact test, p = 0.012).

These findings were confirmed by univariable logistic regression. Compared with the axillary drainage basin, the inguinal drainage basin was associated with significantly higher odds of SLNB positivity (OR 3.31; 95% CI, 1.49–7.35; p = 0.003), whereas no significant difference was observed for the cervical drainage basin (OR 1.58; 95% CI, 0.80–3.10; p = 0.19). The odds of SLNB positivity were also significantly higher in the inguinal than in the cervical drainage basin (OR 2.10; 95% CI, 1.04–4.24; p = 0.04).

**Figure 4:**
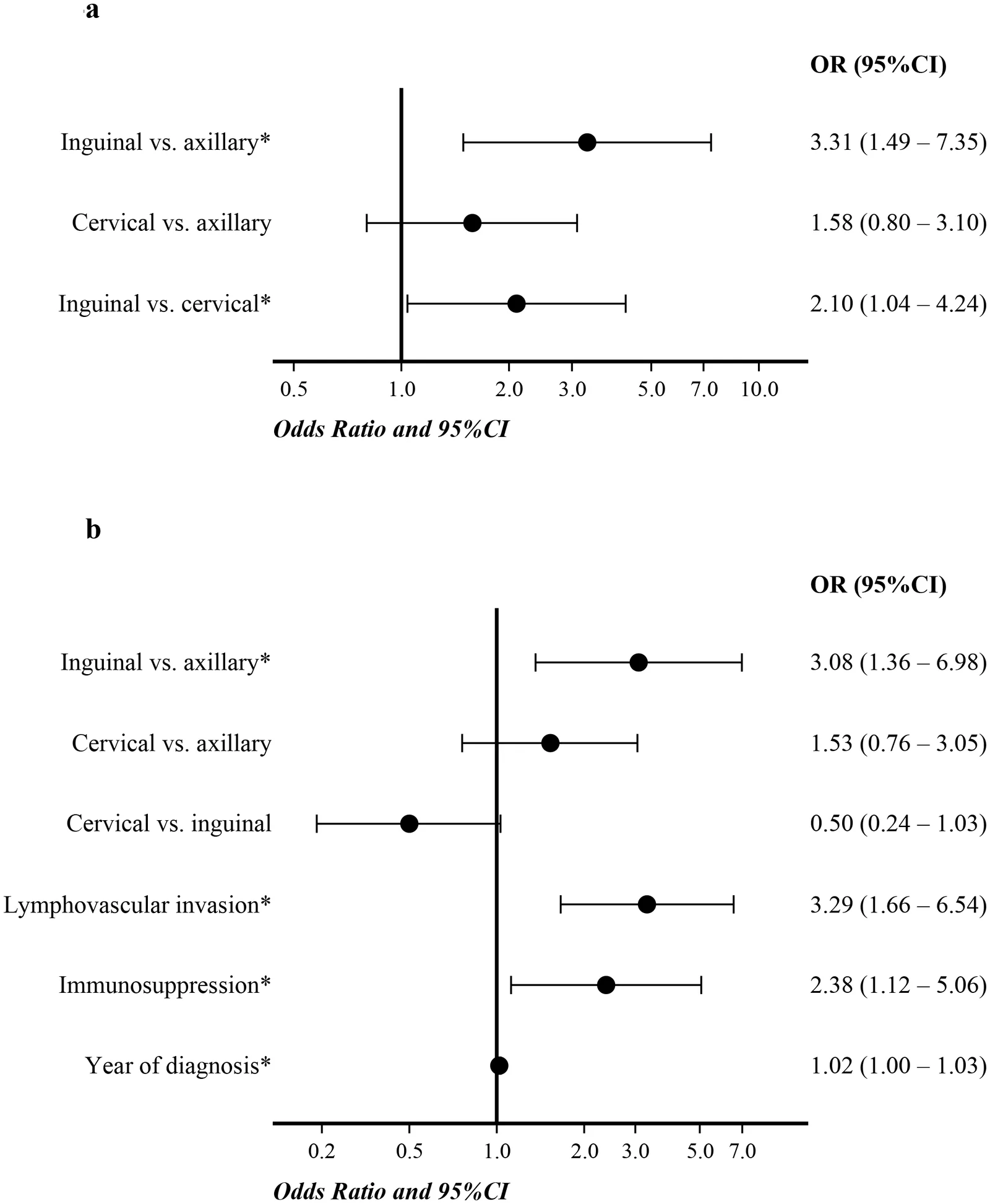
**(a)** Forest plot depicting the unadjusted odds ratios for a positive SLNB across anatomical drainage basins (n = 244; 71 events), from univariable logistic regression. The inguinal basin was associated with significantly higher odds of SLNB positivity than either the axillary or the cervical basin. Circles denote point estimates, horizontal bars 95% confidence intervals, and the vertical line the null value (OR = 1.0). The x-axis is logarithmic. *p < 0.05. **(b)** Forest plot depicting the adjusted odds ratios for a positive SLNB (n = 244; 71 events), from Firth’s penalized logistic regression adjusting for drainage basin, lymphovascular invasion, immunosuppression status, and year of diagnosis. Inguinal drainage remained associated with SLNB positivity after adjustment; *lymphovascular invasion and immunosuppression were also associated with SLNB positivity in the adjusted model, while year of diagnosis showed a modest positive trend.* The year-of-diagnosis estimate is expressed per additional calendar year. The adjusted model showed modest discrimination, with an apparent AUC of 0.70 that decreased to an optimism-corrected AUC of 0.67 (95% CI, 0.59–0.75) after bootstrap validation (500 resamples; mean optimism 0.03). Calibration was adequate (Hosmer–Lemeshow χ^2^[8] = 7.21; p = 0.51). The model is exploratory and hypothesis-generating.

After adjustment for lymphovascular invasion, immunosuppression status, and year of diagnosis using Firth’s penalized logistic regression, inguinal drainage remained associated with SLNB positivity. Compared with axillary SLNB, inguinal SLNB showed significantly higher odds of positivity (OR 3.08; 95% CI, 1.36–6.98; p = 0.007), whereas cervical SLNB did not differ significantly from axillary (OR 1.53; 95% CI, 0.76–3.05; p = 0.23). Cervical SLNB also showed lower odds of positivity than inguinal SLNB, although this did not reach significance (OR 0.50; 95% CI, 0.24–1.03; p = 0.058). Lymphovascular invasion (OR 3.29; 95% CI, 1.66–6.54; p = 0.001) and immunosuppression (OR 2.38; 95% CI, 1.12–5.06; p = 0.025) were also associated with SLNB positivity in the adjusted model, and year of diagnosis showed a modest positive trend (OR 1.02 per year; 95% CI, 1.00–1.03; p = 0.011). A detailed are analysis is depict in **Figure 4**. The adjusted model showed modest discrimination, with an apparent AUC of 0.70 that decreased to an optimism-corrected AUC of 0.67 (95% CI, 0.59–0.75) after bootstrap validation (500 resamples; mean optimism 0.03), and adequate calibration (Hosmer–Lemeshow χ^2^[8] = 7.21; p = 0.51). With 71 events for 5 estimated parameters (approximately 14 events per variable), the model is presented as exploratory and hypothesis-generating.

### Lymph-node dissection

**Figure 5:**
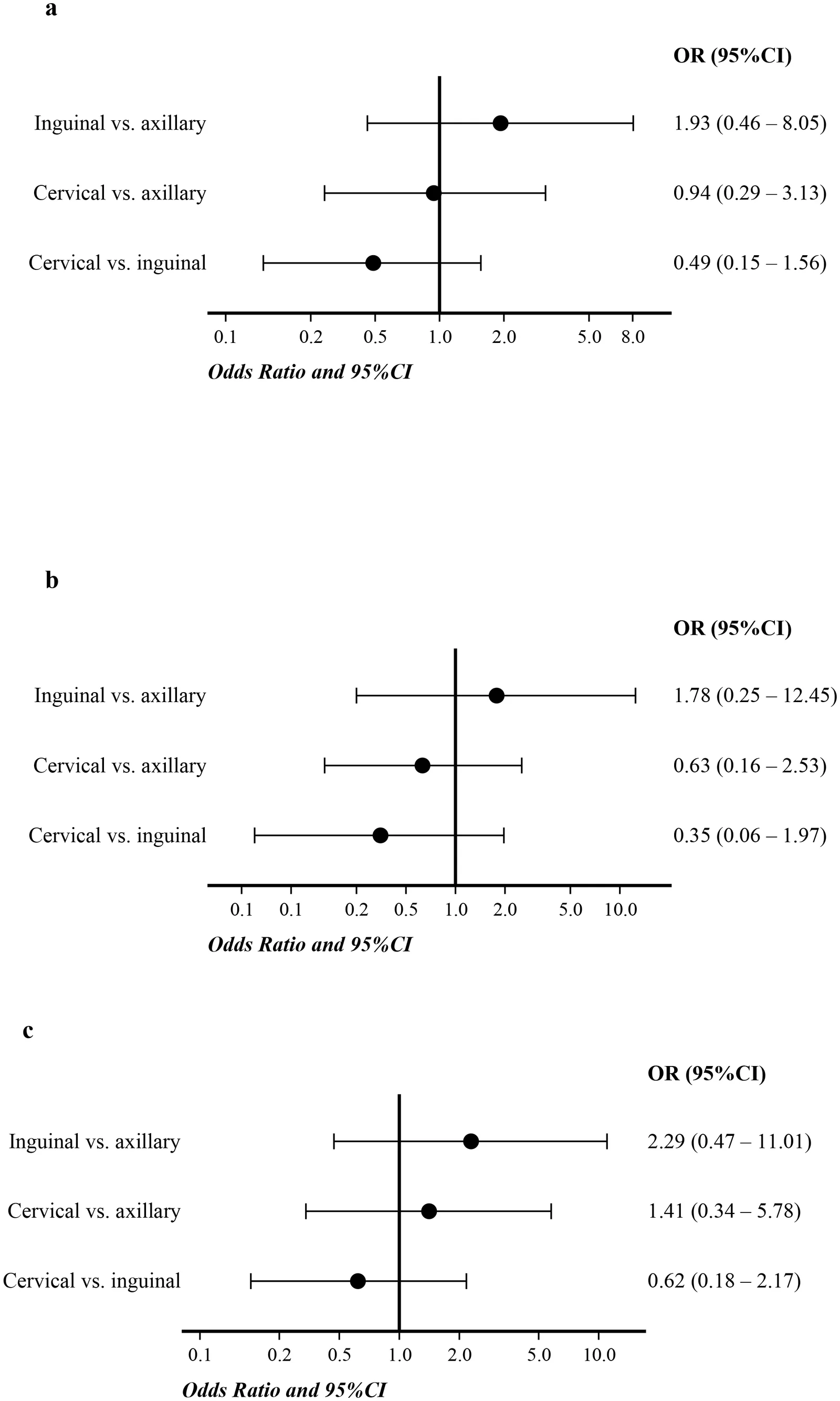
**(a)** Forest plot illustrating the unadjusted odds ratios for the identification of at least one additional metastatic non-SLN at CLND, among patients who underwent SLNB followed by CLND (n = 75; 32 events). No comparison reached statistical significance (Fisher’s exact test, p = 0.47); all confidence intervals cross the null value. Owing to the limited sample size, a multivariable model could not be reliably estimated, and these estimates are hypothesis-generating only. **(b)** Forest plot illustrating the unadjusted odds ratios for at least one histopathologically positive lymph node at primary LND performed without a preceding SLNB, in patients with clinically advanced disease (n = 52; 34 events). No comparison reached statistical significance (Fisher’s exact test, p = 0.50); all confidence intervals cross the null value. Owing to the limited sample size and low statistical power, these estimates are exploratory only. **(c)** Forest plot illustrating the unadjusted odds ratios for the identification of at least one additional metastatic non-SLN at CLND, restricted to patients with a positive SLNB (n = 56; 24 events). No comparison reached statistical significance (Fisher’s exact test, p = 0.55); all confidence intervals cross the null value. These estimates are hypothesis-generating.

Among the 244 patients who underwent SLNB, 75 (30.73%) proceeded to subsequent LND (**Table 3**). Additional metastatic tumor in at least one non-SLN was identified in 32 cases (42.67%): 6 axillary (18.75%), 9 inguinal (28.13%), and 17 cervical (53.13%). The proportion of patients with at least one additional metastatic non-SLN after LND was highest in the inguinal region (9/16; 56.25%), compared with axillary (6/15; 40.00%) and cervical (17/44; 38.64%), although these differences were not statistically significant (p = 0.47). Logistic regression similarly showed no significant associations (inguinal vs. axillary: OR 1.93; 95% CI, 0.46–8.05; p = 0.37; cervical vs. axillary: OR 0.94; 95% CI, 0.29–3.13; p = 0.93; cervical vs. inguinal: OR 0.49; 95% CI, 0.15–1.56; p = 0.23 (**Figure 5a**).

Given the limited sample size of 75 patients, a multivariable adjusted model could not be reliably estimated; results should therefore be interpreted with caution and considered hypothesis-generating only.

### Lymph node dissection in advanced cases

In rare cases with advanced metastatic disease, patients underwent immediate LND without prior SLNB (n = 52). In this cohort, 34 patients (65.38%) had at least one histopathologically positive lymph node. The proportion of patients with at least one histopathologically positive lymph node after primary LND was highest in the inguinal region (80.0%, 8/10), followed by axillary (69.2%, 9/13) and cervical (58.6%, 17/29). These differences were not statistically significant on Fisher’s exact test (p = 0.50) or logistic regression (inguinal vs. axillary: OR 1.78; 95% CI, 0.25–12.45; p = 0.56; cervical vs. axillary: OR 0.63; 95% CI, 0.16–2.53; p = 0.51; cervical vs. inguinal: OR 0.35; 95% CI, 0.06–1.97; p = 0.24) (**Figure 5b**).

Given the limited sample size of 52 patients and the resulting insufficient statistical power, a multivariable adjusted model could not be reliably estimated for this subgroup; these findings should therefore be considered exploratory only.

### Subgroup analysis: patients with positive SLNB

Finally, we explored outcomes in the subgroup of patients with a positive SLNB. Of 244 patients, 71 (29.1%) were SLNB-positive, and 56 of these (78.9%) underwent subsequent CLND. Among them, 24 patients (42.86%) had at least one additional metastatic non-SLN: 4 axillary (16.7%), 8 inguinal (33.3%), and 12 cervical (50.0%). The proportion of patients with affected lymph nodes after CLND was highest in the inguinal region (8/15; 53.33%), compared to cervical (12/29; 41.38%) and axillary (4/12; 33.33%), although these differences were not statistically significant (p = 0.55). Logistic regression similarly showed no significant associations (inguinal vs. axillary: OR 2.29; 95% CI, 0.47 – 11.01; p = 0.30; cervical vs. axillary: OR 1.41; 95% CI, 0.34 – 5.78; p = 0.63; cervical vs. inguinal: OR 0.62; 95% CI, 0.18 – 2.17; p = 0.45).

## Discussion

Although MCC is characterized by both lymphatic and hematogenous dissemination, the present study specifically focused on regional lymphatic spread and nodal staging. In univariable analyses, inguinal drainage basin showed significantly higher SLNB positivity than both axillary and cervical drainage. This association remained significant after adjustment for lymphovascular invasion, immunosuppression, and year of diagnosis, suggesting that inguinal drainage basin remained associated with an increased likelihood of SLNB positivity after adjustment for the available covariates. Importantly, anatomical drainage basin is strongly linked to the location of the primary tumor (head and neck–cervical, upper extremity–axillary, lower extremity–inguinal), although this relationship is not absolute, particularly for tumors of the trunk. Regional comparisons should also be interpreted in the context of the anatomical complexity of the head and neck and potential differences in surgical practice. The persistence of this association after adjustment suggests that additional biological or clinical factors may contribute, although these could not be assessed in the present study.

Previous multivariable studies have identified several independent predictors of SLNB positivity in MCC, including lymphovascular invasion, tumor size, tumor-infiltrating lymphocytes, infiltrative growth pattern, and primary tumor location (5, 17–19). Consistent with these findings, lymphovascular invasion was also associated with SLNB positivity in our cohort. Interestingly, immunosuppression was likewise associated with SLNB positivity in our adjusted model. As this association has not been consistently reported in previous multivariable studies, it should be interpreted cautiously and requires confirmation in independent cohorts. However, previous studies primarily focused on characteristics of the primary tumor, whereas the anatomical lymphatic drainage basin has received little attention (10). Our findings suggest that drainage basin may provide additional information for nodal risk assessment beyond established tumor-related risk factors. To our knowledge, this is the first study to specifically evaluate the association between anatomical drainage basin and SLNB positivity in MCC using multivariable analysis.

The reasons for the observed association between inguinal drainage basin and SLNB positivity remain unclear. Only a few studies have specifically addressed MCC arising in the lower extremities. Scampa et al. reported improved overall survival for lower-extremity MCC and suggested that a higher prevalence of MCPyV-positive tumors in this anatomical region may partly explain these findings (20). More recently, Martin et al. demonstrated that MCPyV-positive MCC occurs more frequently on UV-protected skin than virus-negative disease, while Harms et al. similarly demonstrated that MCPyV-positive MCC represents a biologically distinct subgroup characterized by a lower mutational burden and a more favorable prognosis than virus-negative tumors (21, 22). Together, these observations raise the possibility that biological differences related to viral status may contribute to anatomical variation in MCC behavior. However, MCPyV status was not available in our cohort and therefore could not be evaluated as a potential contributor to the observed regional differences. Gor et al. demonstrated that MCC of the lower extremity and trunk was more frequently diagnosed at advanced stages than head and neck MCC (9). Although this may partly reflect delayed diagnosis because of reduced tumor visibility, this hypothesis has not been confirmed and could not be evaluated in our cohort.

Our findings may be relevant for the management of patients with clinically node-negative MCC. Although anatomical drainage basin alone should not influence treatment decisions, the higher SLNB positivity rate observed in inguinal drainage basins may be considered during preoperative patient counseling and highlights the importance of careful nodal staging in this subgroup. Nevertheless, treatment decisions should always be based on the overall clinicopathological risk profile, rather than drainage basin alone.

A key challenge in the management of MCC is determining the role of CLND after a positive SLNB. Our findings demonstrate that CLND identified additional metastatic lymph nodes in 42.86% of SLNB-positive patients. The high proportion of additional metastatic nodes identified at CLND in our cohort supports the concept that SLNB may underestimate the true nodal tumor burden in a subset of patients, highlighting its diagnostic and staging value. Similarly, Lee et al. reported additional non-SLN metastases in approximately one-third of patients undergoing CLND. Positive non-SLN were associated with significantly poorer oncological outcomes, highlighting the prognostic information provided by CLND (23). Current European and NCCN guidelines therefore continue to recommend further nodal treatment following a positive SLNB, although the optimal therapeutic approach remains under debate (12).

Notably, SLNB was not performed in 39% (156/400) of patients. Of these, one third (52 patients, 33.33%) underwent immediate lymph node dissection due to clinically advanced disease, while the remaining two thirds (104 patients, 66.67%) did not undergo SLNB or LND, most commonly because of alternative treatments such as radiotherapy, refusal of operative therapy, or medical ineligibility related to comorbidities or advanced tumor progression. In advanced cases in which SLNB was not performed, direct LND identified metastatic lymph nodes in 65.38% of patients, reflecting the high nodal tumor burden in clinically node-positive disease.

Although CLND frequently identifies additional metastatic lymph nodes, several contemporary studies have demonstrated comparable locoregional control and survival outcomes for patients treated with nodal radiotherapy alone compared with CLND, particularly when combined with appropriate adjuvant treatment (23–25). Consequently, the present findings primarily support the diagnostic and staging value of CLND rather than its therapeutic superiority. Our study was not designed to evaluate therapeutic efficacy, survival, or recurrence outcomes.

One possible explanation for the additional metastatic lymph nodes identified at CLND is that SLNB examines only the SLN, whereas metastatic disease may already involve non-SLNs within the same drainage basin. Furthermore, despite its high diagnostic accuracy, SLNB is associated with reported false-negative rates of approximately 14–19%, particularly in anatomically complex regions such as the head and neck (14, 26, 27). Metastatic cells may also bypass SLN through alternative lymphatic pathways, resulting in skip metastases. Karunaratne et al. described complex lymphatic drainage patterns, including multiple ipsilateral and contralateral SLN and unpredictable drainage pathways, which may contribute to skip metastases, particularly in the head and neck (28). Although these drainage patterns have been described primarily in the head and neck, they illustrate that lymphatic spread is not always strictly sequential.

Several limitations should be considered when interpreting these findings. First, its retrospective single-center design may introduce biases, such as selection bias or incomplete data, and may limit the generalizability of the results. However, because MCC is a rare malignancy, retrospective cohort studies remain an important source of clinical evidence. Additionally, variability in SLNB practices, including surgical techniques, pathological assessments, and surgeon expertise, may have influenced outcomes and lymph node positivity rates. Although the histopathological diagnosis of MCC has remained well established throughout the study period, LVI classification was based on the original pathology reports without central pathological review or retrospective standardization. As pathological reporting practices may have evolved over the 37-year study period, some degree of misclassification cannot be excluded. Primary tumor size was available only for a subset of patients and was therefore not included in the primary analyses to avoid substantial loss of sample size. As primary tumor size is an established predictor of nodal involvement in MCC, residual confounding cannot be excluded. Variability in lymph node dissection practices, particularly the number of lymph nodes removed across regions such as the cervical, axillary, and inguinal areas, further complicates comparisons. Secondly, the extended inclusion period from 1986 to 2023 inevitably encompasses substantial changes in diagnostic imaging, pathological assessment, staging systems, and systemic treatment strategies. Consequently, temporal treatment heterogeneity may have influenced the observed outcomes. This extended study period also allowed the inclusion of a large cohort of patients with this uncommon disease. Furthermore, important biological factors such as MCPyV status were not available in our cohort, limiting the ability to explore potential biological explanations for the observed regional differences. Although inguinal drainage basin remained associated with SLNB positivity after multivariable adjustment, residual confounding by unavailable clinicopathological variables cannot be excluded. Finally, the lack of long-term survival and recurrence data prevents conclusions regarding the therapeutic benefit of CLND. Consequently, the present findings primarily support the diagnostic and staging value of SLNB and CLND rather than their therapeutic benefit. Future multicenter prospective studies with standardized methodologies and long-term follow-up are needed to validate these findings.

This large single-center cohort study provides further evidence supporting the clinical value of SLNB in the staging of Merkel cell carcinoma and demonstrates a substantial prevalence of occult nodal metastases. While inguinal drainage basins showed higher SLNB positivity rates, inguinal drainage remained associated with nodal involvement after adjustment for clinicopathological factors. CLND identified additional metastatic lymph nodes in a considerable proportion of SLNB-positive patients and remains an important staging tool in selected clinical scenarios. These findings may help inform clinical decision-making regarding nodal staging and the management of regional lymph nodes in patients with MCC. Prospective multicenter studies are warranted to validate these findings and further clarify the mechanisms underlying the observed association between anatomical drainage basin and SLNB positivity. If confirmed, anatomical drainage basin may represent a simple anatomical marker that complements established clinicopathological predictors during nodal risk assessment.

## Data Availability

The original contributions presented in the study are included in the article/supplementary material. Further inquiries can be directed to the corresponding author.
